# The Differential Effects of Attentional Focus in Children with Moderate and Profound Visual Impairments

**DOI:** 10.3389/fpsyg.2017.01804

**Published:** 2017-10-11

**Authors:** Scott W. T. McNamara, Kevin A. Becker, Lisa M. Silliman-French

**Affiliations:** Department of Kinesiology, Texas Woman’s University, Denton, TX, United States

**Keywords:** visual impairment, attentional focus, motor learning, balance, sensory feedback

## Abstract

It has been consistently reported that an external focus of attention leads to better motor performance than an internal focus, but no research to date has explored this effect in a population with visual impairments (VI). External focus statements typically reference something in the environment (e.g., target) that may be difficult to conceptualize for people with VI since they cannot generate a visual representation of the object of focus. Internal focus statements could be more closely identifiable with proprioception that is not impaired in this population. Recent studies have reported that sighted adults with temporarily obstructed vision are able to receive an external focus benefit when performing discrete tasks (i.e., golf putt and vertical jump), however, it is unclear if those with VI would experience the same benefit. The purpose of this investigation was to compare how an internal focus and external focus impact the balance of children with VI. Eighteen children with VI were grouped into a moderate (*n* = 11) and a profound VI group (*n* = 7). Participants completed a familiarization trial, an internal focus trial (i.e., focusing on feet) and an external focus trial (i.e., focusing on markers) in a counterbalanced order. The moderate VI group had a lower root mean square error while using an external focus (*p* = 0.04), while the profound VI group did not differ between conditions (*p* > 0.05). These results suggest that while performing a task reliant on sensory feedback, an external focus benefit may be dependent on the severity of VI. Further research is needed to examine whether external focus statements can be presented in a way that may be more intuitive to those with profound VI. These findings may help to influence how professionals in health-related fields (e.g., physical therapist and physical educators) give instructions on motor performance to populations with VI.

## Introduction

Visual impairment (VI) is defined as a significant impairment in vision and sight, which, even with corrective intervention (e.g., glasses), adversely affects a person’s ability to perform everyday activities (Centers for Disease Control and Prevention, 2011). VI affects around 3% of the United States’ population, with a significant portion being children and adolescents (Brault, 2012). VI is a low incidence disability, which means it occurs less frequently in general populations compared to other disabilities (e.g., learning disabilities; Ludlow et al., 2005). VI can vary by severity and one type of classification was established by the United States Association of Blind Athletes (2016) ^1^ for use in adapted sport. This classification system involves four classes of VI based on severity (see **Table 1**).

**Table 1:** USABA classification system (usaba.org).

USABA classification	USABA definition
B1	No light perception in either eye up to light perception, but inability to recognize the shape of a hand at any distance or in any direction.
B2	From ability to recognize the shape of a hand up to visual acuity of 20/600 and/or a visual field of less than 5° in the best eye with the best practical eye correction.
B3	From visual acuity above 20/600 and up to visual acuity of 20/200 and/or a visual field of less than 20° and more than 5° in the best eye with the best practical eye correction.
B4	From visual acuity above 20/200 and up to visual acuity of 20/70 and a visual field larger than 20° in the best eye with the best practical eye correction.


A lack of balance is one of the most significant deficiencies identified in children with VI (Bouchard and Tetreault, 2000; Brambring, 2006). Those most affected are younger children, and those with profound VI (Rutkowska et al., 2015). This deficiency in balance is a concern as it can increase the risk of falls (Cheung et al., 2008; Ray et al., 2008), and delay or halt the development of locomotor skills (Skaggs and Hopper, 1996; Brambring, 2006; Rutkowska et al., 2015). These combined concerns highlight the importance of identifying and implementing effective instructional strategies that can improve the balance of children with VI.

One potential approach to addressing balance issues in children with VI is to consider the role of cognitive strategies (e.g., attentional focus) associated with improved balance in the typically developing population. Researchers studying focus of attention have consistently demonstrated that an external focus (i.e., focusing on the movement effect) enhances motor performance and learning when compared to an internal focus (i.e., focusing on body movements; Wulf, 2013). This is true across a wide variety of motor tasks including balance tasks (Wulf et al., 1998; Shea and Wulf, 1999; Totsika and Wulf, 2003), object control skills (Zachry et al., 2005), and locomotor skills (Stoate and Wulf, 2011). An external focus has also been reported to improve motor performance in populations with disabling conditions; such as adults with stroke and Parkinson’s disease (Landers et al., 2005) and children with intellectual disabilities (Chiviacowsky et al., 2013). While the external focus benefit is well established, the extant literature in this field has not yet examined this effect in children with VI.

The most widely cited explanation for the external focus benefit is the constrained action hypothesis (Wulf et al., 2001a). Wulf and colleagues suggest that adopting an internal focus leads to the conscious control of actions that should otherwise be controlled automatically, whereas an external focus allows movements to organize on a more automatic level. Evidence supporting the constrained action hypothesis includes an external focus resulting in smaller, more frequent postural adjustments in balancing tasks (McNevin et al., 2003), reduced probe reaction times (Wulf et al., 2001a), and reduced normalized jerk (i.e., smoother movements; Kal et al., 2013). Based on the constrained action hypothesis, attentional focus effects should be independent from vision, but some researchers have suggested vision may work as a mediator by changing gaze patterns in targeting tasks (Hodges and Ford, 2007) and optic flow in balancing tasks (Russell, 2007). Perhaps most interesting, Maurer and Zentgraf (2007) proposed that adopting an external focus may heighten awareness of exteroceptive sensory feedback (e.g., vision and auditory), while an internal focus may encourage engaging interoceptive sensory feedback (e.g., proprioception). Individuals with VI should have no impairment in the use of interoceptive feedback to make postural adjustments under an internal focus (e.g., keeping the feet level), but they may have limited function of exteroceptive feedback under an external focus (e.g., keeping markers level) since vision is impaired.

It is also possible that an external focus could be less effective for children with VI due to a lack of familiarity with objects in the environment. Recently, Maurer and Munzert (2013) demonstrated that the familiarity of a cue is an additional factor influencing motor performance. Specifically, they found that both novice and skilled participants performed better when given familiar cues than unfamiliar cues regardless of focus direction (i.e., internal and external). Many individuals with VI struggle to recognize external objects without touching them. Physical touch can provide some familiarity with an external focus cue, but it is unlikely to be as familiar as a reference to the body.

The influence of vision on attentional focus has recently been tested with blindfolded participants. In these studies, an external focus led to better performance than an internal focus in a vertical jump (Abdollahipour et al., 2016), dart throwing (Sherwood et al., 2014), and golf putting (Land et al., 2013). While these findings suggest vision does not impact attentional focus effects, it is important to note that the tasks used differ from balance in that they rely minimally on sensory feedback. Furthermore, these studies involved a short-term obstruction of vision, rather than having a long-term VI meaning movements could be planned based on previously available visual information. It is important to consider whether the same pattern holds true in a population with VIs. Therefore, the purpose of this study was to compare how an internal and external focus of attention impact the performance of a balancing task in children with VI. Based on the results of previous investigations involving blindfolded typically developing adults, attentional focus effects seem to operate independently of vision, meaning individuals with VI should experience the same benefit as typically developing peers (Land et al., 2013; Abdollahipour et al., 2016). However, if an external focus involves a greater reliance on exteroceptive feedback, or is less familiar to children with VI, they may not receive the same benefit as their typically developing peers.

## Materials and Methods

### Participants and Setting

Convenience sampling was used to recruit participants from a week long overnight summer sports camp for children and youth with VI that was conducted at a university in north Texas. Out of 26 campers, 18 (9 male and 9 female) chose to participate in the study and ranged from ages 9 to 17 (*M* = 12.28, *SD* = 0.71). The study was conducted in a motor behavior laboratory on the campus. The inclusion criteria for participants were: (a) need to have a B1, B2, B3, or B4 visual acuity score, and (b) be between the ages of 8 and 18 years old. USABA vision classification scores (e.g., B2 and B3) were reported by the participant’s parent or guardian. Of the 18 participants, 7 had a B1 or B2 vision score (3 male and 4 female) with an age range between 10 and 17 (*M* = 13.29, *SD* = 2.43); and 11 had a B3 or B4 vision score (6 male and 5 female) with an age range between 9 and 17 (*M* = 11.64, *SD* = 2.25). This study was carried out in accordance with the recommendations of the Texas Woman’s University Institutional Review Board. Written informed consent was obtained from each participant’s parent or guardian at the registration of the camp, and verbal assent was obtained from each participant prior to initiating data collection in accordance with the Declaration of Helsinki. For data analysis purposes and because of the limited sample size, participants were grouped into two categories rather than the four classifications based on the USABA classification system. The two groups were the profound VI group (i.e., B1 or B2 vision acuity) or moderate VI group (i.e., B3 or B4 vision acuity).

### Task and Apparatus

**FIGURE 1:**
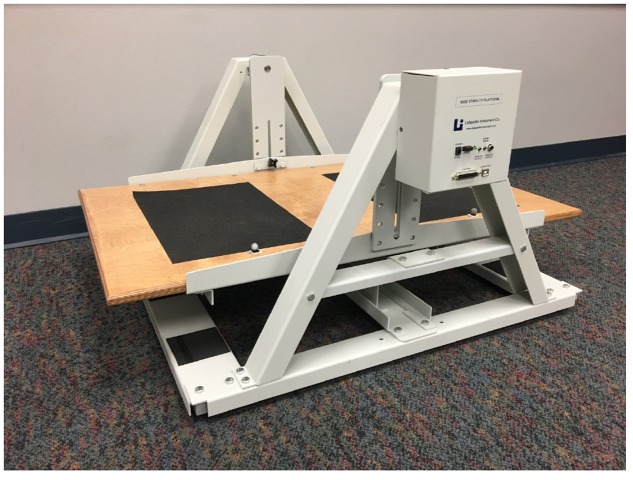
Stability platform.

The task involved standing on a Stability Platform (Lafayette Instruments Model 16030, Lafayette, IN, United States), and making postural adjustments to keep the platform as level as possible. The stability platform and the software to analyze the angles of the platform have been used in numerous previous attentional focus studies (e.g., Shea and Wulf, 1999; Wulf et al., 2001a). The apparatus consists of a wooden platform (42 cm × 25.5 cm) attached to a support structure by two freely rotating axles (see **Figure 1**). Two small spherical markers were placed on the platform in front of where the participants’ feet would be placed. These markers were used for external focus instructions. The maximum possible deviation of the platform was 15° in either direction. The stability platform was connected to a standard PC (HP Elitebook), and data were collected and processed with Psymsoft 2 software (Lafayette Instruments, Lafayette, IN, United States).

### Procedures

Prior to the initiation of the actual investigation, a separate pilot study with two children with VI was conducted to ensure the researchers consistency with implementing the experimental tasks and data collection, as well as, to ensure the task was appropriate for children with VI. Participants were escorted from the camp activity to the lab one at a time by a research assistant. On arrival to the lab, the researchers introduced the participant to the experimental task and procedures. Each participant completed one familiarization trial and two experimental trials. Each trial consisted of balancing on the platform for 60 s followed by 60 s of rest. The two experimental trials (i.e., internal and external focus) were presented in a counterbalanced order, and participants were randomly assigned to an order prior to arriving in the lab. Before each trial, the participants gave verbal assent that they were ready for the trial and felt safe. During all trials, a research assistant stood on each side of the platform and acted as a spotter. For the familiarization trial, participants were allowed to hold the hands of the research assistants until they felt comfortable, but were instructed that they would need to balance on their own during experimental trials.

Prior to each experimental trial, participants were instructed to focus on a particular statement throughout the entire trial. For the internal focus trial, participants were told “On this trial we want you to focus on keeping your *feet* level,” and on the external focus trial, they were told “On this trial we want you to focus on keeping the *markers* on the platform level.” Prior to starting the external focus trials, participants were asked to physically touch each of the markers to be sure they were aware of their location and shape. Additionally, during all trials a focus reminder was given every 20 s to maximize compliance with the focus statements. For all trials, participants were instructed to look straight ahead to avoid a confounding difference of visual focus. On completion of the two experimental trials, each participant was asked “What condition did you feel you performed better at and why do you feel this way?” and was then escorted back to the camp activities by a research assistant.

### Data Analysis

The Psymsoft 2 software was used to record the position of the stability platform at intervals of every 0.04 s throughout each trial. These data points were used to calculate root mean square error (RMSE), which was the primary dependent variable. RMSE was calculated with a zero-degree reference. A 2 × 2 mixed model ANOVA was used to test for main effects and interactions related to attentional focus (i.e., internal and external) and vision level (i.e., profound VI and moderate VI). Sidak *post hoc* tests were used to detect the source of significant interactions. Partial η^2^ values are reported as effect sizes, with values interpreted as small (0.01–0.09), medium (0.09–0.25), and large (greater than 0.25; Carlson and Winquist, 2013). The alpha level for all analyses was set at 0.05.

## Results

### Root Mean Square Error

**FIGURE 2:**
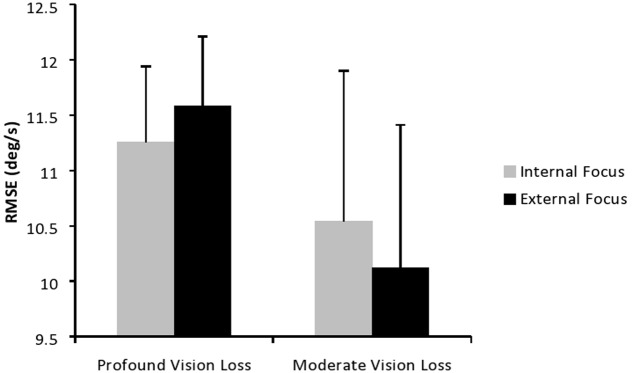
Root mean square error with an internal and external focus of attention.

Displayed in **Figure 2** are the means and standard deviations of participants’ RMSE while performing the task with an internal and external focus. Results of the mixed model ANOVA indicated the main effect of vision approached significance, *F*(1,16) = 4.38, *p* = 0.053, $\eta_{p}^{2}$ = 0.22, with children in the moderate group (*M* = 10.33, *SD* = 0.33) trending toward lower error scores than those in the profound group (*M* = 11.42, *SD* = 0.41). The main effect of focus failed to reach significance (*p* > 0.05), but the interaction between vision and focus was significant, *F*(1,16) = 6.05, *p* = 0.03, $\eta_{p}^{2}$ = 0.27. Sidak *post hoc* tests were used to detect the source of the interaction, and indicated that participants with moderate VI had significantly lower error with an external focus (*M* = 10.12, *SD* = 0.33) than an internal focus (*M* = 10.54, *SD* = 0.35; *p* = 0.04). However, those with profound VI did not differ between focus types (*p* > 0.05).

### Questionnaire Feedback

Displayed in **Table 2** are the representative statements from the question, “What condition did you feel you performed better at and why do you feel this way?” Participants’ answers were categorized into two groups: the profound VI group and the moderate VI group. In the profound VI group five out of seven participants reported a perceived internal focus advantage. In the moderate VI group six perceived an internal focus advantage while the other five perceived an external focus advantage.

**Table 2:** Open-ended questionnaire responses.

VI group	Question 1: (a) What condition did you feel you performed better at and (b) why do you feel this way?
Moderate VI	(a) Feet (i.e., internal), (b) because when you asked me to focus on the markers (i.e., external), I didn’t know how to keep them level, but I knew how to keep my feet level.
	(a) Markers, I don’t know, I just like when I try to even it, I just think about my feet, sometimes I put more weight on one then the other, but if I think about the markers I am less worried about putting the right pressure.
	(a) Focusing on my feet (b) because that is easier to picture rather than the two little markers.
Profound VI	(a) Feet, (b) I felt like I could sort of, um, I guess I was more perceptive. Because it is something I can physically feel the markers. The markers are something I cannot see, so I feel like it is harder to keep them level. Because you use your feet on a daily basis to keep you balance, using your feet to keep you balanced is something you can understand more.
	(a) It was harder to focus on the markers, (b) because they are so small, they can move very easy. Because I walk everyday and my feet are a part of me, I can look down at my feet.
	(a) Markers,
	(b) because my feet I can’t really tell if they are level, but the markers I can kind of visualize that they need to be straight up.


## Discussion

The benefits of an external focus of attention have been well established in the literature (Wulf, 2013), but it is unclear if children with VI would experience the same benefit when performing a balancing task. Recent research has demonstrated that when participants are blindfolded and performing discrete tasks, an external focus improves motor performance (Land et al., 2013; Sherwood et al., 2014; Abdollahipour et al., 2016). However, the same pattern may not hold true for tasks more reliant on sensory feedback, especially in participants with VI who are accustomed to performing motor tasks with limited vision or a complete absence of vision. The purpose of this study was to determine how an internal focus and external focus of attention affect the balance performance of children with VI.

Based on the results of the present investigation, it seems the benefit of an external focus of attention may be dependent on the severity of VI. Children with moderate VI (i.e., classified as B3 and B4) had less balance error when using an external focus, which supports the constrained action hypothesis by suggesting that when vision is moderately impaired, an external focus can still result in more automated processing. This finding coincides with the widespread results from previous research with typically developing adults and typically developing children (Wulf et al., 2010; Wulf, 2013; Perreault and French, 2016). In addition to balance error data, previous research involving the same task has reported increased mean power frequency (MPF) (i.e., smaller, more frequent adjustments) while using an external focus of attention (Wulf et al., 2001a). In that study, Wulf and colleagues did not report MPF values during acquisition due to difficulty interpreting the values when participants “slammed” the platform on the base going back and forth from left to right early in acquisition. Due to the performance nature of the present study, we encountered similar issues and therefore decided to report only RMSE as a dependent variable of interest.

An interesting result of this study is that those with profound VI (i.e., B1 and B2) did not experience an external focus benefit. In interpreting these results, it is important to first consider the characteristics of those with profound VI. Participants with a B1 VI range from being completely blind to having light perception, while those with a B2 VI are able to detect objects, but within a very limited field of vision (i.e., less than 5°). These participants typically use a cane for locomotion, and require substantial assistance for daily life activities. Maurer and Zentgraf’s (2007) proposal about attentional focus and vision may offer an intriguing explanation of this finding, but has limitations due to the design of the experiment. They suggested that an external focus encourages more automatic processing by relying on exteroceptive feedback (i.e., vision) to make movement adjustments. Conversely, they proposed that an internal focus encourages engaging with interoceptive feedback resulting in more conscious control of limbs and muscles responsible for producing the movements. While this may partially explain the results from this study, the participants were instructed to look straight ahead in the experiment, meaning direct visual feedback of the marker position should have been outside the visual field of any participants regardless of the level of VI. Exteroceptive feedback related to body position could have aided participants with higher levels of vision in achieving or maintaining balance, but it was unlikely to have provided feedback about marker position.

A more plausible explanation of the effects here relates to the familiarity of focus statements. Previous research indicates that in addition to focus direction (i.e., external and internal), the familiarity of focus statements has an influence on performance (Maurer and Munzert, 2013). In the present study, the external focus cue “keep the markers level” may have been less familiar to participants with profound VI than the internal statement “keep your feet level” due to the inability to see what the markers looked like. Physical guidance was used to give participants information about the location and shape of the markers prior to engaging in the external focus trial, however, they touched the markers for only a few seconds in a static position. Without a strong mental representation of what the markers looked like when in a dynamic state, these participants may have found the external focus statement difficult to use. Questionnaire results present some evidence this may have been the case as five out of seven of the profound VI participants perceived that an internal focus was more effective than an external focus in maintaining balance.

Previous researchers have also suggested participants vary in their preference for either an internal or external focus (Wulf et al., 2001b; Weiss et al., 2008; Marchant et al., 2009; Maurer and Munzert, 2013). Participants in some studies predominantly prefer an external focus (Wulf et al., 2001b; Marchant et al., 2009), while others report a frequent internal preference (Maurer and Munzert, 2013). Weiss et al. (2008) tested whether an interaction between focus direction and preference existed, and found that performance deteriorated when those who preferred an external focus were forced to use an internal focus. In contrast, those who preferred an internal focus and were allowed to use it saw no performance decrement. Within the small sample of this study, it is possible that an overwhelming preference for an internal focus could have eliminated the benefit of an external focus in the group with profound VI. This preference could be related to an internal focus being more closely aligned with the sensory feedback available to them, but it may also be perpetuated by a bias related to the prominence of internally focused instruction in fields within kinesiology, such as physical education (Fronske and Wilson, 2002), physical therapy (Johnson et al., 2013), and physiotherapy (Durham et al., 2009). Further research should investigate how preference of an internal and external focus affects children with VI’s ability to receive the associated benefits.

The rationale participants provided for why they believed one type of focus was superior was insightful. One participant in the moderate VI group indicated performing with an external focus was easier because when he used an internal focus sometimes he would put too much pressure on one foot. When talking about an external focus, he was “… less worried about putting the right pressure.” This account seems consistent with the constrained action hypothesis (Wulf et al., 2001a), suggesting that he may have achieved more automatic processing under an external focus. In contrast, another moderate VI participant reported an internal focus being more beneficial to the ability to balance “… because that is easier to picture rather than the two little markers.” This comment aligns with Maurer and Munzert’s (2013) findings that familiarity can outweigh the influence of focus direction. A participant from the profound VI group indicated an internal focus being more effective “Because it is something I can physically feel. The markers are something I cannot see, so I feel like it is harder to keep them level.” For this participant, it appears an external focus may be difficult to use when only proprioceptive feedback is available for making postural adjustments. An internal focus, though it may have its limitations was far easier for that participant to conceptualize and use. However, another participant with profound VI had a very different viewpoint in suggesting an external focus was more effective because “… my feet I can’t really tell if they are level, but the markers I can kind of visualize that they need to be straight up.” The variability present in responses suggests that finding the optimal individual attentional focus for each participant may be more difficult than selecting an internally or externally focused cue. The findings of Weiss et al. (2008) and Maurer and Munzert (2013) suggest that a method of allowing participants to sample a variety of cues and select a preferred or familiar cue may lead to the greatest performance.

Limitations of this investigation should be recognized. The first limitation is VI is a low incidence disability, and as a result there was only a small sample size available within both groups (i.e., moderate and profound). Replicating this study with a larger sample size, particularly with those with profound VI would be very useful in understanding what type of attentional focus is most effective for them since they tend to have the most impaired balance. A second limitation is that in attentional focus research it is impossible to directly control a participant’s attentional focus. Frequent reminders, standardized procedures, and compliance checks were used to maximize adherence, but there is always the potential that a participant may use a focus that is different from what was prescribed.

The benefits of an external focus of attention have been well established in the literature, but based on the results of the present investigation, it seems those benefits may be dependent on the severity of VI. It is unclear at this point, however, if the difference relates mechanistically to visual function or if lower vision is associated with differences in familiarity with and preference for certain instructions. Further research is needed to understand the role of vision in attentional focus effects and how that might influence people with VI performing motor skills in real life settings. Replication of the present investigation with a larger VI population would increase generalization of these findings, and allow for identification of familiarity and preference as factors due to increased statistical power. In addition, researchers should examine attentional focus effects in participants with VI using other motor tasks (e.g., throwing) that have been reported to be impacted with typically developing peers (Zachry et al., 2005; Ong et al., 2010; Southard, 2011). With continued research, future and current practitioners who teach children with various degrees of VI may be able to adapt their instruction to include instructions and feedback that align with a focus that maximizes performance and learning.

## Author Contributions

SM developed the research design, collected the data, and was the primary author of the manuscript. KB collaborated closely with the first author and mentored the first author. Specifically, he helped to create the research design, collected data, and provided content and edits to the manuscript. LS-F helped in identifying and securing participants needed for the study and gave edits throughout the creation of the manuscript.

## Conflict of Interest Statement

The authors declare that the research was conducted in the absence of any commercial or financial relationships that could be construed as a potential conflict of interest.

## Footnotes

1
www.usaba.org

## References

[B1] Abdollahipour R. Psotta R. Land W. M. (2016). The influence of attentional focus instructions and vision on jump height performance. *Res. Q. Exerc. Sport* 87 408–413. 10.1080/02701367.2016.1224295 27636712

[B2] Bouchard D. Tetreault S. (2000). The motor development of sighted children and children with moderate low vision aged 8–13. *J. Vis. Impair. Blind.* 94 64–73.

[B3] Brambring M. (2006). Divergent development of gross motor skills in children who are blind or sighted. *J. Vis. Impair. Blind.* 100 620–634.

[B4] Brault M. W. (2012). *United States Economics and Statistics Administration, United States Bureau of the Census: Americans with Disabilities: 2010. Current Population Reports*. Available at: https://www.census.gov/content/dam/Census/library/publications/2012/demo/p70-131.pdf.

[B5] Carlson K. A. Winquist J. R. (2013). *An Introduction to Statistics: An Active Learning Approach*. Thousand Oaks, CA: Sage Publications.

[B6] Centers for Disease Control and Prevention (2011). *Blindness and Vision Impairment.* Atlanta: Centers for Disease Control and Prevention.

[B7] Cheung K. K. Au K. Y. Lam W. W. Jones A. Y. (2008). Effects of a structured exercise programme on functional balance in visually impaired elderly living in a residential setting. *Hong Kong Physiother. J.* 26 45–50. 10.1016/s1013-7025(09)70007-7

[B8] Chiviacowsky S. Wulf G. Avila L. T. G. (2013). An external focus of attention enhances motor learning in children with intellectual disabilities. *J. Intellect. Disabil. Res.* 57 627–634. 10.1111/j.1365-2788.2012.01569.x 22563795

[B9] Durham K. Van Vliet P. M. Badger F. Sackley C. (2009). Use of information feedback and attentional focus of feedback in treating the person with a hemiplegic arm. *Physiother. Res. Int.* 14 77–90. 10.1002/pri.431 19107706

[B10] Fronske H. Wilson R. (2002). *Teaching Cues for Basic Sport Skills for Elementary and Middle School Students.* San Francisco, CA: Benjamin Cummings Publishing Company.

[B11] Hodges N. J. Ford P. (2007). “Skillful attending, looking and thinking,” in *Attentional Focus and Motor Learning [Target Article]* Vol. 1 eds Hossner E.-J. Wenderoth N. Wulf G. (Champaign, IL: Human Kinetics), 23–24.

[B12] Johnson L. Burridge J. H. Demain S. H. (2013). Internal and external focus of attention during gait re-education: an observational study of physical therapist practice in stroke rehabilitation. *Phys. Ther.* 93 957–966. 10.2522/ptj.20120300 23559523

[B13] Kal E. C. Van der Kamp J. Houdijk H. (2013). External attentional focus enhances movement automatization: a comprehensive test of the constrained action hypothesis. *Hum. Mov. Sci.* 32 527–539. 10.1016/j.humov.2013.04.001 24054892

[B14] Land W. M. Tenenbaum G. Ward P. Marquardt C. (2013). Examination of visual information as a mediator of external focus benefits. *J. Sport Exerc. Psychol.* 35 250–259. 10.1123/jsep.35.3.250 23798588

[B15] Landers M. Wulf G. Wallmann H. Guadagnoli M. (2005). An external focus of attention attenuates balance impairment in patients with Parkinson’s disease who have a fall history. *Physiotherapy* 91 152–158. 10.1016/j.physio.2004.11.010

[B16] Ludlow B. L. Conner D. Schechter J. (2005). Low incidence disabilities and personnel preparation for rural areas: current status and future trends. *Rural Spec. Educ. Q.* 24 15–24. 10.1177/875687050502400303

[B17] Marchant D. C. Clough P. J. Crawshaw M. Levy A. (2009). Novice motor skill performance and task experience is influenced by attentional focusing instructions and instruction preferences. *Int. J. Sport Exerc. Psychol.* 7 488–502. 10.1080/1612197x.2009.9671921

[B18] Maurer H. Munzert J. (2013). Influence of attentional focus on skilled motor performance: performance decrement under unfamiliar focus conditions. *Hum. Mov. Sci.* 32 730–740. 10.1016/j.humov.2013.02.001 23830490

[B19] Maurer H. Zentgraf K. (2007). “On the how and why of the external focus learning advantage,” in *Attentional Focus and Motor Learning [Target Article]* Vol. 1 eds Hossner E.-J. Wenderoth N. Wulf G. (Champaign, IL: Human Kinetics), 31–32.

[B20] McNevin N. H. Shea C. H. Wulf G. (2003). Increasing the distance of an external focus of attention enhances learning. *Psychol. Res.* 67 22–29. 12589447 10.1007/s00426-002-0093-6

[B21] Ong N. T. Bowcock A. Hodges N. J. (2010). Manipulations to the timing and type of instructions to examine motor skill performance under pressure. *Front. Psychol.* 1:196. 10.3389/fpsyg.2010.00196 21833255 PMC3153805

[B22] Perreault M. E. French K. E. (2016). Differences in children’s thinking and learning during attentional focus instruction. *Hum. Mov. Sci.* 45 154–160. 10.1016/j.humov.2015.11.013 26638048

[B23] Ray C. T. Horvat M. Croce R. Mason R. C. Wolf S. L. (2008). The impact of vision loss on postural stability and balance strategies in individuals with profound vision loss. *Gait Posture* 28 58–61. 10.1016/j.gaitpost.2007.09.010 18023185

[B24] Russell D. M. (2007). “Attentional focus on the invariant control variables,” in *Attentional Focus and Motor Learning [Target Article]* Vol. 1 eds Hossner E.-J. Wenderoth N. Wulf G. (Champaign, IL: Human Kinetics), 47–48.

[B25] Rutkowska I. Bednarczuk G. Molik B. Morgulec-Adamowicz N. Marszałek J. Kaźmierska-Kowalewska K. (2015). Balance functional assessment in people with visual impairment. *J. Hum. Kinet.* 48 99–109. 10.1515/hukin-2015-0096 26834878 PMC4721628

[B26] Shea C. H. Wulf G. (1999). Enhancing motor learning through external-focus instructions and feedback. *Hum. Mov. Sci.* 18 553–571. 10.1016/S0167-9457(99)00031-7

[B27] Sherwood D. E. Lohse K. R. Healy A. F. (2014). Judging joint angles and movement outcome: shifting the focus of attention in dart-throwing. *J. Exp. Psychol.* 40 1903–1914. 10.1037/a0037187 24911011

[B28] Skaggs S. Hopper C. (1996). Individuals with visual impairments: a review of psychomotor behavior. *Adapt. Phys. Activ. Q.* 13 16–26. 10.1123/apaq.13.1.16 12192500

[B29] Southard D. (2011). Attentional focus and control parameter: effect on throwing pattern and performance. *Res. Q. Exerc. Sport* 82 652–666. 10.5641/027013611x13275192111709 22276407

[B30] Stoate I. Wulf G. (2011). Does the attentional focus adopted by swimmers affect their performance? *Int. J. Sports Sci. Coach.* 6 99–108. 10.1260/1747-9541.6.1.99

[B31] Totsika V. Wulf G. (2003). The influence of external and internal foci of attention on transfer to novel situations and skills. *Res. Q. Exerc. Sport* 74 220–232. 10.1080/02701367.2003.10609084 12848235

[B32] United States Association of Blind Athletes (2016). *Visual Classifications.* Colorado Springs, CO: United States Association of Blind Athletes.

[B33] Weiss S. M. Reber A. S. Owen D. R. (2008). The locus of focus: the effect of switching from a preferred to a non-preferred focus of attention. *J. Sports Sci.* 26 1049–1057. 10.1080/02640410802098874 18608831

[B34] Wulf G. (2013). Attentional focus and motor learning: a review of 15 years. *Int. Rev. Sport Exerc. Psychol.* 6 77–104. 10.1080/1750984x.2012.723728

[B35] Wulf G. Chiviacowsky S. Schiller E. Ávila L. T. G. (2010). Frequent external focus feedback enhances motor learning. *Front. Psychol.* 1:190. 10.3389/fpsyg.2010.00190 21833250 PMC3153799

[B36] Wulf G. Höß M. Prinz W. (1998). Instructions for motor learning: differential effects of internal versus external focus of attention. *J. Mot. Behav.* 30 169–179. 10.1080/00222899809601334 20037032

[B37] Wulf G. McNevin N. Shea C. H. (2001a). The automaticity of complex motor skill learning as a function of attentional focus. *Q. J. Exp. Psychol. A* 54 1143–1154. 10.1080/02724980143000118 11765737

[B38] Wulf G. Shea C. Park J.-H. (2001b). Attention and motor performance: preferences for and advantages of an external focus. *Res. Q. Exerc. Sport* 72 335–344. 10.1080/02701367.2001.10608970 11770783

[B39] Zachry T. Wulf G. Mercer J. Bezodis N. (2005). Increased movement accuracy and reduced EMG activity as the result of adopting an external focus of attention. *Brain Res. Bull.* 67 304–309. 10.1016/j.brainresbull.2005.06.035 16182938

